# Editorial: Neurodegeneration: from Genetics to Molecules

**DOI:** 10.3389/fncel.2016.00187

**Published:** 2016-08-03

**Authors:** Marco A. Meraz-Ríos, Rosalinda Guevara-Guzmán, Karla G. Carvajal, Victoria Campos-Peña

**Affiliations:** ^1^Departamento de Biomedicina Molecular, Centro de Investigación y de Estudios AvanzadosMexico City, Mexico; ^2^Departamento de Fisiología, Facultad de Medicina, Universidad Nacional Autónoma de MéxicoMexico City, Mexico; ^3^Laboratorio de Nutrición Experimental, Instituto Nacional de PediatríaMexico City, Mexico; ^4^Laboratorio Experimental de Enfermedades Neurodegenerativas, Instituto Nacional de Neurología y NeurocirugíaMexico City, Mexico

**Keywords:** neurodegeneration, memory impairment, systemic inflammation and neurodegeneration, dopaminergic neurotransmission, caloric restriction, sirtuins, miRNAs, neuroprotective effects

This research topic gives an overview of the current knowledge on some of the most common processes in neurodegenerative diseases such as Alzheimer, Parkinson and Temporal Lobe Epilepsy. Here we reviewed different aspects of each neurodegenerative process, including epigenetics, oxidative and inflammatory responses, genetic susceptibility, aging, metals and environmental exposure, autophagy, and micro RNA basic research.

Neurodegeneration: from genetics to molecules, is a multidisciplinary research topic aiming to be of interest to basic researchers in medical fields.

Many expected that the splendid and remarkable success of the human genome project introduced toward the end of the Twentieth century would halt the epidemic of neurodegenerative diseases. Yet, rather than diminished, it has risen to become a serious problem of morbidity, loss of useful life years, and mortality worldwide. Simultaneously, laboratory research has continued to unravel successive layers of the pathophysiology of neurodegenerative diseases and the mechanisms of its clinical complications, suggesting new routes to follow for the development of innovative therapeutic approaches.

Hence, the need for a compendium of articles that reviews the current state-of-the-art of neurodegeneration research spanning from a global perspective to fundamental molecular mechanisms. The editors intended this collection of articles in order to highlight the emerging challenges to many tenets of belief that have emerged from recent studies. We do so in the spirit of inspiring future research to attack the unresolved questions and to exploit the newer discoveries that have opened unanticipated horizons of understanding and raised novel questions and opportunities for therapies.

Calero et al. commence the research topic with an update of additional mechanisms conferring genetic susceptibility to Alzheimer's disease. They describe the analysis of mutations in the genes involved in the production of the amyloid related to EOFAD. In addition, they review the genes associated to LOAD and the list of genes associated with sporadic AD. Further, they analyze recent whole-exome studies looking for strong association to the disease, and integrate several studies dealing with epistasis as additional mechanisms conferring genetic susceptibility to AD, searching for networks rather than the contribution of specific genes (Calero et al).

Landgrave-Gómez et al. reviewed the role of epigenetic mechanisms in the function and homeostasis of the central nervous system and their participation in a variety of neurological disorders. The evidence suggests that long-term changes in gene transcription could be associated with changes in chromatin structure and participate in a variety of neurological disorders (Landgrave-Gomez et al).

Chin-Chan et al. evaluated the environmental pollutants as risk factors for Alzheimer (AD) and Parkinson diseases (PD). They discuss the role of environmental factors such as lead, mercury, aluminum, cadmium and arsenic, as well as some pesticides and metal-based nanoparticles in the development of idiopathic AD and PD, and their mechanisms of action.

Ramos-Chávez et al. reviewed the neurological effects of inorganic arsenic (iAs) exposure as an important natural pollutant. They investigate the effects of gestational iAs exposure and evaluate the expression of the cysteine/glutamate transporters in cortex and hippocampus, demonstrating significant differences in spatial memory impairment in males while the effect was marginal in females (Chin-Chan et al).

Sankowski et al. examined the relationship of systemic inflammation and the brain, looking for novel roles of genetic, molecular, and environmental cues as drivers of neurodegeneration. The CNS responds to somatic and autonomic sensory information, also receiving input from the periphery about inflammation and infection. The hypothesis is that the resulting translocation of inflammatory mediators can interfere with neuronal and glial homeostasis. Here, they review recent genetic evidence suggesting an association between neurodegenerative disorders and persistent immune activation and evaluate their potential relevance in neurodegenerative disorders (Sankowski et al).

Joshi and Pratico reviewed the 5-lipoxygenase (5LO) and its downstream leukotriene metabolites as oxidative and inflammatory contributions to Alzheimer's disease. 5LO pathway and 5LO activating protein (FLAP) have been implicated in the molecular pathology of AD, including the metabolism of amyloid-β and tau. In this article you will find an overview of 5LO and FLAP, discussing their involvement in biochemical pathways relevant to AD pathogenesis and how targeting these proteins could lead to therapies relevant not only for AD, but also other related neurodegenerative conditions (Joshi and Pratico).

Toral-Rios et al. evaluated the relationship of polymorphisms of inflammation-related genes with Alzheimer's disease. Since it has been demonstrated that inflammation contributes to the process of neurodegeneration and therefore being a key factor in the development of AD, the authors evaluate the differences in frequencies between AD and controls by single-nucleotide polymorphism (SNP) of inflammation-related genes (Toral-Rios et al).

James et al. analyzed aged neuronal nitric oxide knockout mice. The animals show preserved olfactory learning suggesting that lack of NO releases protected animals against age-associated cognitive decline in memory tasks typically involving olfactory and hippocampal regions, but not against declines in reversal learning or locomotor activity (James et al).

Blanco Ayala et al. studied an alternative kynurenic acid synthesis route in the rat cerebellum. The Kynurenic acid (KYNA) is an endogenous antagonist of α7 nicotinic acetylcholine and excitatory amino acid receptors. Their results suggest that different mechanisms are involved in KYNA production in the rat cerebellum and that, specifically, DAAO and ROS can function as alternative routes for KYNA synthesis (Blanco Ayala et al).

Phillips-Farfán et al. demonstrated that caloric restriction (CR) has a protective effect against amygdala electrical kindling by inhibiting the mTOR signaling pathway. They investigated whether CR altered the levels of insulin and energy substrates. And found that CR decreases protein kinase B phosphorylation and ribosomal protein S6, suggesting an inhibition of the mTOR cascade as well as an increasing after-discharge threshold tending to reduce the after-discharge duration. It suggests an anti-convulsive action which could imply that CR has an anti-epileptic effect via inhibition of the mTOR pathway (Phillips-Farfan et al).

Lucas et al. found cerebellar transcriptional alterations with Purkinje cell dysfunction and loss in mice lacking the transcriptional coactivator peroxisome proliferator-activated receptor γ coactivator-1α (ppargc1a or PGC-1α). The authors found that mice lacking PGC-1α exhibit ataxia in addition to deficits in motor coordination. Their data suggest that dysfunction in multiple cell types contributes to motor deficits in the context of PGC-1α deficiency (Lucas et al).

Braidy et al. evaluated the differential expression of sirtuins in aging rat brains. They tested mRNA and protein expression levels of rat SIRT1-7, and levels of associated proteins in the brain using RT-PCR and western blotting. This article identifies important unknown roles for sirtuins in regulating cellular homeostasis and healthy aging (Braidy et al).

da Luz et al. found that dopamine induces the accumulation of insoluble prion protein and affects autophagic flux. Since dopamine metabolism generates several oxidative metabolites and can be highly reactive, the authors investigated whether these molecules can also affect the aggregation of cellular prion protein (PrPC). Their results bring new insight into the dopamine metabolism as a source of endogenous metabolites capable of altering PrPC solubility and its subcellular localization (da Luz et al).

Rocha et al. studied the GABAergic alterations in neocortex of patients with pharma-co-resistant temporal lobe epilepsy and explain the comorbidity of anxiety and depression. Their results showed a dysfunction of the GABAergic neurotransmission in temporal neocortex of patients with Temporal Lobe Epilepsy and comorbid anxiety and/or depression that could be also influenced by clinical factors such as seizure frequency and duration of illness (Rocha et al).

Mielcarek et al. evaluated histone deacetylase 4 as a potential therapeutic target in neurodegenerative diseases. HDAC4 is a member of the class IIa family of histone deacetylases and is the major player in synaptic. In this review the authors integrate the information regarding the biological role of HDAC4 in neurodegenerative processes (Mielcarek et al).

Carballo-Molina and Velasco evaluated the hydrogels as scaffolds and delivery systems to enhance axonal regeneration after injuries. As hydrogels are biodegradable, biocompatible and have the capacity to deliver a large range of molecules *in situ*, they have been used in several biomedical applications. This review discusses areas of opportunity where hydrogels can be applied, in order to promote axonal regeneration of the NS, by applying hydrogels in combination with cultured neural cells, forming three-dimensional structures, showing the formation of synapses and neuronal survival (Carballo-Molina and Velasco).

Garza-Manero et al. performed the identification of age- and disease-related alterations in circulating miRNAs in a mouse model of Alzheimer's disease. In this study the authors evaluated the levels of miRNA at different time-points in a transgenic mouse model of Alzheimer disease (3xTg-AD). They found age-related significant changes in miRNA abundance for both WT and transgenic mice. Some of these were specific for the 3xTg-AD, suggesting that the age-dependent evolution of the AD-like pathology, rather than the presence and expression of the transgenes, modifies the circulating miRNA levels in the 3xTg-AD mice (Garza-Manero et al).

Correa-Basurto et al. using molecular dynamics and docking simulations, realized the identification of the antiepileptic racetam binding site in the synaptic vesicle protein 2A (SV2A). SV2A is the molecular target of the anti-epileptic drug levetiracetam and its racetam analogs. The importance of this study is that identifying the racetam binding site within SV2A will facilitate the synthesis of new radio-ligands to improve treatment response and evaluate epilepsy progression (Correa-Basurto et al).

Acquarone et al. studied in a mouse model of Parkinson's disease (PD), mitomycin-treated undifferentiated embryonic stem cells as a safe and effective cellular therapeutic strategy. The authors show that undifferentiated mouse embryonic stem cells (mESCs), pre-treated with mitomycin C (MMC) before transplantation, prevented tumorigenesis in a 12 week follow-up after mESCs were injected in nude mice and in 6-OH-dopamine-lesioned mice. Intrastriatal injection of MMC-treated mESCs markedly improved motor function without tumor formation. These findings show novel strategies for cell therapies and particularly for treatment of PD (Acquarone et al).

González-Castillo et al. reviewed the role of pleiotrophin (PTN) as a central nervous system (CNS) neuromodulator. In this paper, the authors highlight and summarize the most recent advances and results that lead to proposing a PTN as a neuromodulatory molecule in the CNS, particularly in the hippocampus. Interesting data in CNS demonstrate that PTN exerts post-developmental neurotrophic and -protective effects, and recently has been involved in neurodegenerative diseases and neural disorders (Gonzalez-Castillo et al).

Gonzalez-Lima et al. provided an update on the cellular mechanisms mediating the neuroprotective effects of low doses of methylene blue and near-infrared light. In addition the authors demonstrate how the neurotherapeutic benefits of these two different interventions share the same cellular mechanism of action. Highlighting the similarities in energy transfer, low-dose hormetic dose-responses, and enhanced capacity for oxidative metabolic energy production (Gonzalez-Lima and Auchter).

Szabadfi et al. analyzed the retinal aging phenomena in the diurnal Chilean rodent (*Octodon degus*). Their results show overexpression of GFAP in Müller glial cells in aging retinas and a reduction in the number of rod bipolar cells and the ganglion cells while that of cone bipolar cells remained unchanged. Their results are very interesting adding novel data to understand the retinal aging process (Szabadfi et al).

Finally, we recognized that a single collection of articles cannot deal with the extremely large number of topics that characterize a complex multifactor condition such as neurodegeneration. The topics addressed, however, help developing a clear idea, not only of what has been obtained to date by previous studies but also of the unmet needs future research should focus on. We trust that the papers assembled in this research topic will prove useful in spurring and stimulating this future progress.

## Author contributions

The author MM and VC prepared the Editorial. All authors: VC, MM, RG, and KC contributed significantly to the review and editing of each manuscript accepted on this topic.

### Conflict of interest statement

The authors declare that the research was conducted in the absence of any commercial or financial relationships that could be construed as a potential conflict of interest.

